# The Adductomics of Isolevuglandins: Oxidation of IsoLG Pyrrole Intermediates Generates Pyrrole–Pyrrole Crosslinks and Lactams

**DOI:** 10.3390/ht8020012

**Published:** 2019-05-10

**Authors:** Wenzhao Bi, Geeng-Fu Jang, Lei Zhang, John W. Crabb, James Laird, Mikhail Linetsky, Robert G. Salomon

**Affiliations:** 1 Department of Chemistry, Case Western Reserve University, Cleveland, OH 44106, USA; wxb96@case.edu (W.B.); jxl122@case.edu (J.L.); mdl78@case.edu (M.L.); 2 Cole Eye Institute, Cleveland Clinic Foundation, Cleveland, OH 44195, USA; jangg@ccf.org (G.-F.J.); zhangl@ccf.rg (L.Z.); crabbj@ccf.org (J.W.C.)

**Keywords:** adductome, covalent adducts, electrophilic species, mass spectrometry, reactive metabolites, isolevuglandins, protein crosslinking

## Abstract

Isoprostane endoperoxides generated by free radical-induced oxidation of arachidonates, and prostaglandin endoperoxides generated through enzymatic cyclooxygenation of arachidonate, rearrange nonenzymatically to isoprostanes and a family of stereo and structurally isomeric γ-ketoaldehyde seco-isoprostanes, collectively known as isolevuglandins (isoLGs). IsoLGs are stealthy toxins, and free isoLGs are not detected in vivo. Rather, covalent adducts are found to incorporate lysyl ε-amino residues of proteins or ethanolamino residues of phospholipids. In vitro studies have revealed that adduction occurs within seconds and is uniquely prone to cause protein–protein crosslinks. IsoLGs accelerate the formation of the type of amyloid beta oligomers that have been associated with neurotoxicity. Under air, isoLG-derived pyrroles generated initially are readily oxidized to lactams and undergo rapid oxidative coupling to pyrrole–pyrrole crosslinked dimers, and to more highly oxygenated derivatives of those dimers. We have now found that pure isoLG-derived pyrroles, which can be generated under anoxic conditions, do not readily undergo oxidative coupling. Rather, dimer formation only occurs after an induction period by an autocatalytic oxidative coupling. The stable free-radical TEMPO abolishes the induction period, catalyzing rapid oxidative coupling. The amine N-oxide TMAO is similarly effective in catalyzing the oxidative coupling of isoLG pyrroles. N-acetylcysteine abolishes the generation of pyrrole–pyrrole crosslinks. Instead pyrrole-cysteine adducts are produced. Two unified single-electron transfer mechanisms are proposed for crosslink and pyrrole-cysteine adduct formation from isoLG-pyrroles, as well as for their oxidation to lactams and hydroxylactams.

## 1. Introduction

Modification of Proteins by Isolevuglandins

**Scheme 1:**
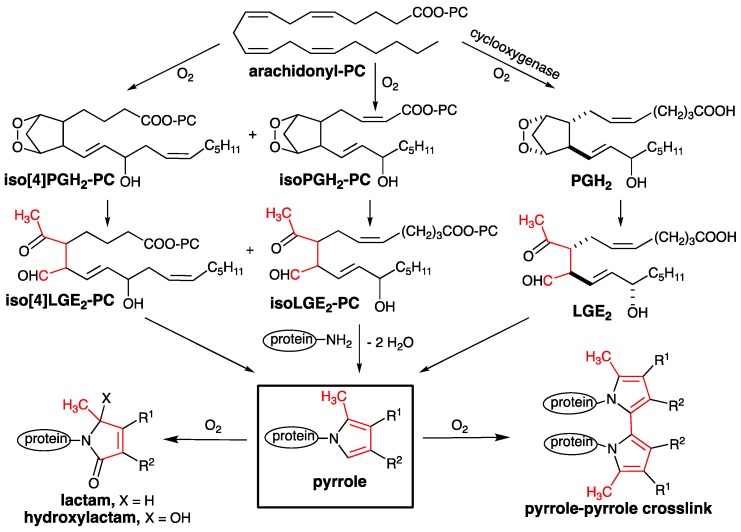
Biosynthesis of isolevuglandins and their covalent adduction to proteins.

The prostaglandin endoperoxide intermediate PGH_2_ of the cyclooxygenase pathway, and its stereo and structural isomers, e.g., the isoprostane endoperoxides isoPGH_2_ or iso[4]PGH_2_, which are generated as phospholipid esters through free radical-induced oxidation of arachidonyl phospholipids, spontaneously rearrange to produce various stereo and structurally isomeric γ-ketoaldehydes (Scheme 1), referred to collectively as isolevuglandins (isoLGs). Covalent adduction of isoLGs to proteins occurs within seconds. The adducts include derivatives of isoLGs that incorporate the ε-amino group of protein lysyl residues in a pyrrole ring. These electron-rich pyrroles are readily oxidized to form lactams and hydroxylactams that are the major isoLG derivatives of proteins in vivo. Covalent adduction of isoLGs also generates protein–protein crosslinks within minutes [1]. We recently demonstrated that one type of crosslink is a pyrrole–pyrrole dimer generated by the oxidative coupling of isoLG-derived pyrroles (Scheme 1) [2].

The adduction of isoLG with other biomolecules, that forms pyrroles and more oxidized derivatives, has been linked to alcoholic liver disease, Alzheimer’s disease, age-related macular degeneration, atherosclerosis, cardiac arythmias, cancer, end-stage renal disease, glaucoma, inflammation of allergies and infection, mitochondrial dysfunction, multiple sclerosis, and thrombosis [3]. The first peptide mapping and sequencing of an isoLG-modified protein present in human retina identified the modification of a specific lysyl residue of the sterol C27-hydroxylase Cyp27A1 [4]. This residue is preferentially modified by iso[4]LGE_2_ in vitro, causing a loss of function.

Because the crosslinking of proteins in vivo is likely to have pathological consequences, e.g., accelerating the formation of the type of oligomers of amyloid beta that has been associated with neurotoxicity [5], we sought to acquire insights into the chemistry of isoLG-derived pyrroles. We now report that the pure isoLG-derived pyrrole, isolated from the reaction of iso[4]LGE_2_ with the dipeptide acetyl-gly-lys-O-methyl ester under anoxic conditions, does not readily undergo oxidative coupling when incubated in air. A slow autocatalytic dimerization does occur upon exposure to air. We also report that the oxidative dimerization of isoLG-derived pyrroles is blocked by N-acetylcycteine which intercepts a putative pyrrole cation radical intermediate to produce isoLG pyrrole-cysteine crosslinks. Finally, trimethylamine-N-oxide (TMAO), the accumulation of which is strongly positively correlated with risk of cardiovascular disease [6], catalyzes the oxidation of isoLG-pyrrole to form crosslinks, lactams, and hydroxylactams.

## 2. Materials and Methods

General Methods

All of the chemicals used were high-purity analytical grade. The following commercially available materials were used as received: the acetyl-gly-lys-O-methyl ester was from Bachem (Torrance, CA, USA), all other chemicals were obtained from Fisher Scientific Co., (Chicago, IL, USA). HPLC was performed with HPLC-grade solvents.

MALDI-TOF Analyses

MALDI-TOF mass spectrometry was performed on an AB Sciex4800 Plus MALDI TOF/TOF™ Analyzer (AB Sciex LLC, Framingham, MA, USA), equipped with a UV laser (355 nm) and reflector mode with a matrix of α-cyano-4-hydroxycinnamic acid (CHCA, 10 mg/mL in acetonitrile/water/3% formic acid, 5:4:1, v/v/v). The plate was calibrated with peptide mass standards. Volatiles were evaporated from reaction aliquots that were resuspended in 10% aqueous acetonitrile solution and vortexed to mix well before C18 Ziptip purification (Millipore Sigma, Burlington, MA, USA, ZipTip with 0.6 μL C18 resin). To wet the C18 Ziptip, the pipettor plunger was depressed to a dead stop using the maximum volume setting of 20 μL. Acetonitrile (wetting solution) was aspirated into the ZipTip and dispensed to waste three times. The tip was equilibrated for binding by washing three times with the 0.1% formic acid/water (equilibration solution). Samples were bound to the ZipTip by fully depressing the pipettor plunger to a dead stop, then aspirating and dispensing the sample for 3 to 7 cycles. The tip was then washed with at least 3 cycles of 0.1% formic acid/water (wash solution) dispensing to waste. The samples were then eluted with 5 μL of 80% acetonitrile/0.1% formic acid (elution solution) into a clean vial to give a final concentration of 100 fmol/μL by aspirating and dispensing eluant through the ZipTip at least three times without introducing air. Two microliters of the sample solution were then mixed with 1 μL of matrix. 1.5 μL were applied to the plate spot and allowed to dry. Data was processed with AB Sciex Data Explorer 4.0 software (AB Sciex LLC, Framingham, MA, USA.).

Preparation of Iso[4]LGE_2_-pyrrole

N-Acetyl-gly-lys methyl ester (2 mg, 7.71 μmol) was added to 1 mL distilled H_2_O and the solution was sparged with argon for several minutes to remove air. Then, Na_2_S_2_O_4_ (1 mg, 5.75 μmol) was added to this solution. Iso[4]LGE_2_ in methanol (136 μg, 0.386 μmol, 5 μg/μL) was added and the mixture was incubated overnight at room temperature. Thin layer chromatography showed the presence of unreacted dipeptide, only one major product, and the complete disappearance of iso[4]LGE_2_. Water was removed from the solution by transfer under high-vacuum into a dry ice acetone-cooled trap. Then, methanol (1 mL) was added to dissolve the organic product. The solution was loaded onto a 6 cm column in a disposable Pasteur pipette packed with a slurry of silica gel in EtOAc, and eluted with methanol (2 mL) (R_f_ = 0.5) to remove the bulk of the sodium dithionite. After concentration under reduced pressure, deaerated reagent-grade chloroform (1 mL), which had been sparged with argon, was added to dissolve iso[4]LGE_2_-pyrrole and the solution was filtered to remove the remaining traces of sodium dithionite and any silical gel that had dissolved in the methanol. The solution of crude iso[4]LGE_2_-pyrrole (~222 μg, 0.39 μmol) was concentrated to dryness by evaporation of solvents with a stream of argon, and the residue was dissolved in 10% acetonitrile/water (500 μL). 

Iso[4]LGE_2_-pyrrole was purified by HPLC with a 2.1 × 100 mm^2^ i.d C18 1.8 μm column (Waters Acquity, UPLC HSS). Chromatography was carried out with linear elution gradient (elute A, 0.1% (v/v) formic acid (FA)/H_2_O; elute B, 0.1% (v/v) FA/acetonitrile (ACN)) at a flow rate of 150 μL/min. To determine the retention time of iso[4]LGE_2_-pyrrole, ~222 μg (0.39 μmol), which had been freed of Na_2_S_2_O_4_, was dissolved in 10% (v/v) ACN 0.1% (v/v) FA (50 mL) and then loaded onto the column with 20% eluate B for 10 min and eluted with the following gradient: 20–60% B over 20 min, to 98% within 0.1 min, and hold for 5 min. The effluent was monitored by ESI-MS/MS with an API-3000 triple quadrupole electrospray mass spectrometer (Applied Biosystems Inc.). The instrument was operated in the positive mode, and high-pressure nitrogen was used as source gas, and scanning from m/z 220–2000 was performed using the parameters listed in Table S1. The pyrrole peak eluted between 12.8 and 14 min. Another aliquot of ~222 mg was injected and the eluent was collected in 1 min fractions from 11 to 31 min and the fractions were analyzed for purity by MALDI-TOF MS. Fractions containing the pure pyrrole were combined. Optimized parameters for the triple quadrupole mass spectrometer are listed in Table S2.

Autoxidation of Iso[4]LGE_2_-pyrrole

Purified iso[4]LGE_2_-pyrrole (45 μg, 0.078 μmol in 450 μL 10% (v/v) acetonitrile/water) was incubated under air at 25 °C on a shaker (IKA MTS 2/4 digital microtiter shaker, 500 rpm). At various time points (2, 6, and 8 days), aliquots (15 μg, 0.026 μmol in 150 μL 10% acetonitrile/water) from the reaction mixture were quickly dried using a high-speed vacuum evaporator and analyzed by MALDI-TOF MS.

Autoxidation of Iso[4]LGE_2_-pyrrole in the Presence of N^α^-acetylcysteine

A solution of N^α^-acetylcysteine (7.7 μL of 100 mM), containing 0.77 μmol (126 μg) in H_2_O that had been neutralized by the addition of NaOH, was added to purified iso[4]LGE_2_-pyrrole (44.36 μg, 0.077 μmol in 500 μL 10% (v/v) acetonitrile/water) and the mixture was incubated under air at 25 °C on a shaker (IKA MTS 2/4 digital microtiter shaker, 500 rpm). In a control experiment, an identical mixture was incubated under argon at 25 °C on a shaker (IKA MTS 2/4 digital microtiter shaker, 500 rpm). In another control experiment, an identical mixture was incubated under argon in the presence of dithionite at 25 °C on shaker (IKA MTS 2/4 digital microtiter shaker, 500 rpm). At various time points (2, 4, 6, and 8 days), aliquots of the reaction mixtures (0.019 μmol, 125 μL) were quickly dried using a high-speed vacuum evaporator and analyzed by MALDI-TOF MS. 

Autoxidation of Iso[4]LGE_2_-pyrrole in the Presence of APS/TEMED

Ammonium persulfate (APS, 1.6 μL of 1 mM in H_2_O, 1.544 nmol, 352.4 ng) and tetramethylethylenediamine (TEMED, 0.7 μL of 1 mM in H_2_O, 0.772 nmol, 90 ng) were added to iso[4]LGE_2_-pyrrole (44 μg, 0.077 μmol in 500 μL 10% (v/v) acetonitrile/water) and incubated under air on a shaker (IKA MTS 2/4 digital microtiter shaker, 500 rpm). At various time points (1 h, 2 h, 3 h, and 1 day) an aliquot of the reaction mixture (0.019 μmol, 125 μL) was quickly dried using a high-speed vacuum evaporator and analyzed by MALDI-TOF MS. 

Autoxidation of Iso[4]LGE_2_-pyrrole in the Presence of TMAO or TEMPO

Trimethylamine-N-oxide (TMAO, 7.7 μL of 1 mM in pH 7 H_2_O, 7.7 nmol, 5.8 μg) was added to HPLC-purified iso[4]LGE_2_-pyrrole (44 μg, 0.077 μmol in 500 μL 10% (v/v) acetonitrile/water) and the mixture was incubated under air on a shaker (IKA MTS 2/4 digital microtiter shaker, 500 rpm). At various time points (1 h, 2 h, 3 h, and 1 day), aliquots of the reaction mixture (0.019 μmol, 125 μL) were quickly dried using a high-speed vacuum evaporator and analyzed by MALDI-TOF MS. The same experiment was performed with (2,2,6,6-tetramethylpiperidin-1-yl)oxyl (TEMPO, 1 mM in 10% ACN, pH 6) in place of TMAO. 

## 3. Results

### 3.1. Preparation of Iso[4]LGE_2_-Pyrrole under Anoxic Conditions

**Scheme 2:**
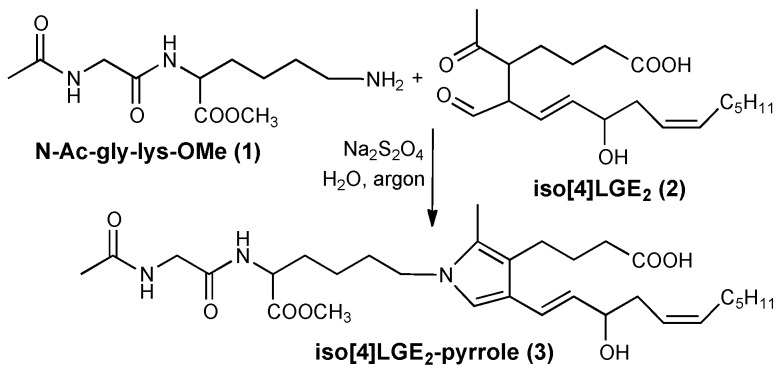
Synthesis of iso[4]LGE_2_-pyrrole.

**Figure 1:**
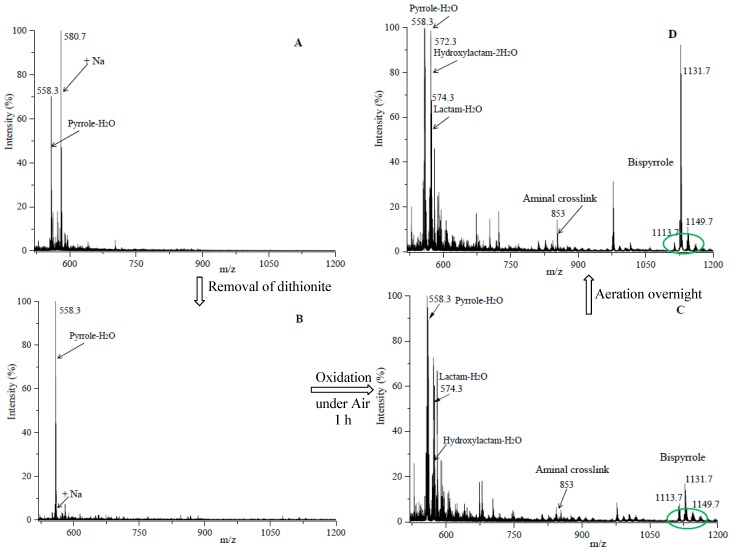
MALDI-TOF MS analysis of iso[4]LGE_2_-pyrrole (**3**) and oxidation products from the reaction mixture from iso[4]LGE_2_ (**2**) with N-acetyl-glycyl-lysine methyl ester (**1**) in the presence of dithionite after exposure to air or aeration with oxygen before or after removal of dithionite. (**A**) Reaction mixture from N-acetylglycyllysine methyl ester (**1**) and iso[4]LGE_2_ (**2**, molar ratio = 20:1) in the presence of sodium dithionite under an atmosphere of argon at 25 °C overnight. (**B**) Pure iso[4]LGE_2_-pyrrole (**3**) immediately after removing the sodium dithionite. (**C**) Reaction mixture of pure iso[4]LGE_2_-pyrrole (**3**) after 1 h exposure to air. (**D**) Reaction mixture of pure iso[4]LGE_2_-pyrrole (**3**) after bubbling O_2_ overnight.

As reported previously, the formation of pyrrole–pyrrole crosslinks was detected upon adduction of isoLGs with N-acetyl-gly-lys methyl ester in air [2]. Because pyrrole–pyrrole dimer formation is an oxidative process, crosslinking is not expected to occur under anoxic conditions. In pilot studies, we found that incubation of N-acetyl-gly-lys methyl ester with iso[4]LGE_2_ under an atmosphere of argon in aqueous solution that had been sparged with argon before and during reaction, was not sufficient to exclude all traces of oxygen. Besides iso[4]LGE_2_-pyrrole (**3**), products of further oxidation, i.e., lactam and hydroxylactam, were also generated (data not shown). To preclude formation of pyrrole–pyrrole dimer, lactam, or hydroxylactam, a synthesis of **3** was achieved under rigorously anoxic conditions. This was achieved by conducting the adduction in the presence of sodium dithionite (Na_2_S_2_O_4_), an oxygen scavenger that can maintain the oxygen concentration at extremely low levels [7]. Incubation of iso[4]LGE_2_ (**2**) with an excess of N-acetyl-gly-lys methyl ester (1, 20 equivalents) to favor complete consumption of the γ-ketoaldehyde, generated the adduct iso[4]LGE_2_-pyrrole (Scheme 2) with no products of further oxidation, as assessed by MALDI analysis (Figure 1A).

To remove sodium dithionate from the reaction mixture, water was removed and methanol was added to dissolve the organic product. Then the mixture was loaded onto a column, packed with a slurry of silica gel in EtOAc, and eluted with methanol. After removal of the methanol under reduced pressure, deaerated chloroform, which had been sparged with argon, was added to dissolve iso[4]LGE_2_-pyrrole and the solution was filtered to remove any remaining traces of sodium dithionite and silica gel that had dissolved in the methanol. Evaporation of the solvent under a stream of argon delivered a mixture containing iso[4]LGE_2_-pyrrole and excess N-Ac-gly-lys-OMe uncontaminated with lactam, hydroxylactam, or pyrrole–pyrrole dimer. MALDI analysis of a solution of this mixture in 10% acetonitrile/water immediately after removing the sodium dithionite showed no oxidation products (Figure 1B). As expected, exposure of this product to air in the absence of dithionate for one hour produced various oxidized derivatives of iso[4]LGE_2_-pyrrole (**3**), e.g., lactam, hydroxy­lactam, and bispyrrole (Figure 1C). Bubbling oxygen through the solution overnight generated more of these oxidation products, and the bispyrrole became a major product (Figure 1D). 

**Figure 2:**
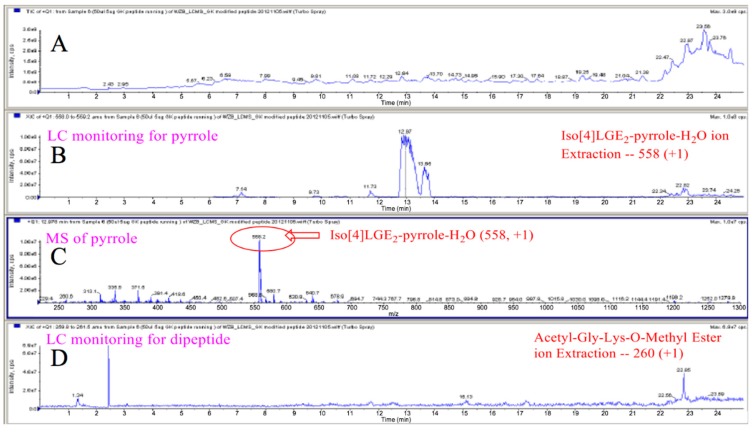
Liquid chromatography-electrospray ionization monitoring of the purification of iso[4]LGE_2_-pyrrole with selected ion recording for iso[4]LGE_2_-pyrrole. (**A**) Total Ion Current, (**B**,**C**) LC monitoring and MS analysis for iso[4]LGE_2_-pyrrole-H_2_O (*m/z* 558 (+ H^+^)), (**D**) LC monitoring for N-acetyl-gly-lys methyl ester (*m/z* 260 (+ H^+^)).

Since excess acetyl-gly-lys methyl ester and other impurities might influence its susceptibility to autoxidation, iso[4]LGE_2_-pyrrole was purified by HPLC with an acetonitrile/water gradient using LC-ESI to detect the components of interest by monitoring the appropriate molecular mass ions in the extracted ion channels (Figure 2). The fraction eluting between 12.8 and 14 min contained pure iso[4]LGE_2_-pyrrole (**3**). The absence of oxidized pyrrole or bispyrrole products [2] in UPLC-purified iso[4]LGE_2_-pyrrole was confirmed by MALDI-TOF MS (Figure 1B and Supplementary Information Figure S1). The structure is consistent with the presence, in the MALDI-TOF spectrum of the HPLC pure fraction, of a prominent peak corresponding to the expected loss of H_2_O. The formation of isoLG-pyrrole adducts was established previously by conversion to a stable triflouroacetylated derivative that was fully characterized by ^1^H and ^13^C NMR as well as high-resolution mass spectroscopy [8]. MALDI and triple quadrupole mass spectrometry analyses also confirmed that this sample of pure iso[4]LGE_2_-pyrrole was not contaminated with unreacted N-acetyl-gly-lys methyl ester or iso[4]LGE_2_. The background of the matrix for MALDI-TOF and the solvent used in triple quadrupole mass spectrometry was determined to not exhibit any peaks corresponding to N-acetyl-gly-lys methyl ester or iso[4]LGE_2_ in the matrix or solvent (no m/z around 260 (+1 for N-acetyl-gly-lys methyl ester) or 353 (+1 for iso[4]LGE_2_)). Peaks not corresponding to the matrix or solvent peaks, which were not present in the sample of pure iso[4]LGE_2_ pyrrole (**3**), could be readily detected by MALDI or triple quadrupole mass spectrometric analysis of N-acetyl-gly-lys methyl ester or iso[4]LGE_2_ (see Supplementary Information Figure S2). 

### 3.2. An Electron Transfer Mechanism for Pyrrole–Pyrrole Crosslinking by Oxygen

**Scheme 3:**
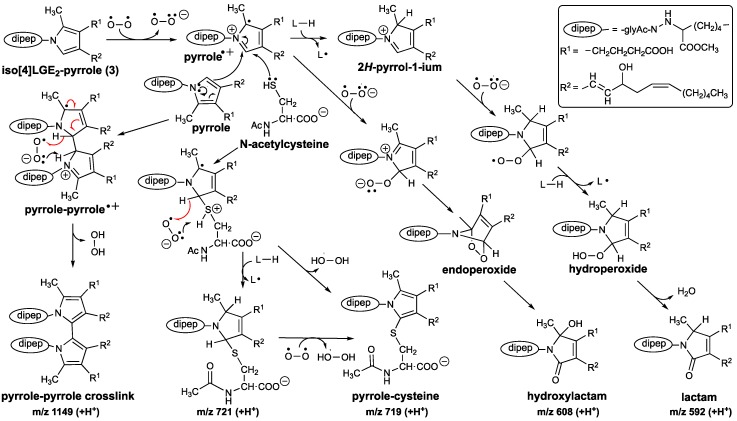
A mechanism involving single electron transfer from iso[4]LGE_2_-pyrrole to oxygen for pyrrole–pyrrole oxidative coupling, oxidation to lactam and hydroxylactam derivatives and oxidative coupling with N-acetylcysteine. Single electron movement is indicated with red half-headed arrows.

We postulated that the oxidative coupling of pyrroles by molecular oxygen with concomitant formation of lactam and hydroxylactams might occur by a single electron transfer mechanism [9] involving electrophilic aromatic substitution with pyrrole as a nucleophile and a pyrrole cation radical as an electrophile (Scheme 3). This mechanism predicts that the intermediate cation radical might be intercepted by other nucleophiles, e.g., a thiol.

### 3.3. Reaction of Iso[4]LGE_2_-Pyrrole with N^α^-acetylcysteine

**Scheme 4:**
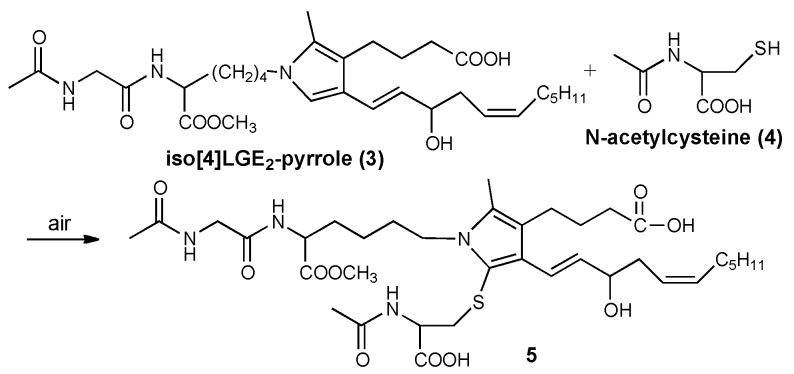
Reaction of pure iso[4]LGE_2_-pyrrole with N-acetylcysteine.

**Figure 3:**
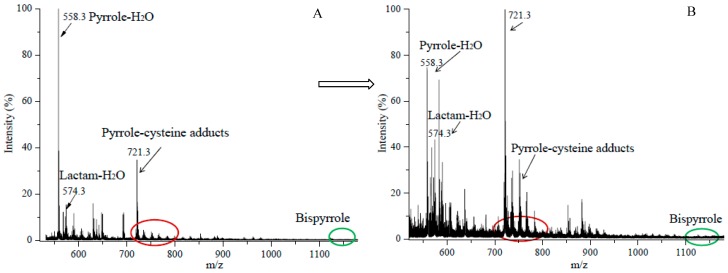
MALDI-TOF MS spectra of pure iso[4]LGE_2_-pyrrole reaction with N-acetylcysteine under air. (**A**) Reaction mixture of pure iso[4]LGE_2_-pyrrole and N-acetylcysteine (molar ratio = 1:10) at 25 °C for 2 days. (**B**) Reaction mixture of pure iso[4]LGE_2_-pyrrole and N-acetylcysteine (molar ratio = 1:10) at 25 °C for 8 days.

**Figure 4:**
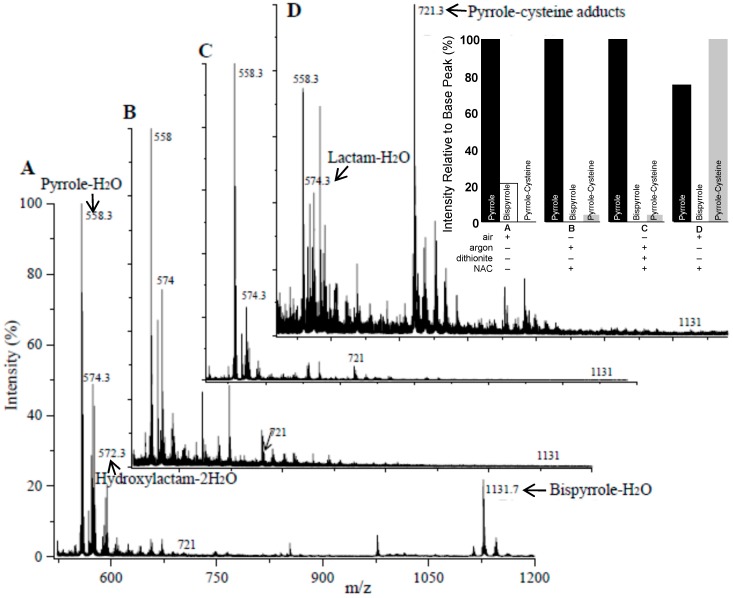
MALDI-TOF MS of pure iso[4]LGE_2_-pyrrole and its reaction with N-acetylcysteine. Trace A = pure iso[4]LGE_2_-pyrrole incubation under air at 25 °C for 8 days. Trace B = pure iso[4]LGE_2_-pyrrole reaction with N-acetylcysteine under argon at 25 °C for 8 days. Trace C = pure iso[4]LGE_2_-pyrrole reaction with N-acetylcysteine under argon in the presence of sodium dithionite at 25 °C for 8 days. Trace D = pure iso[4]LGE_2_-pyrrole reaction with N-acetylcysteine under air at 25 °C for 8 days. The bar graph compares levels of three products in A–D.

**Figure 5:**
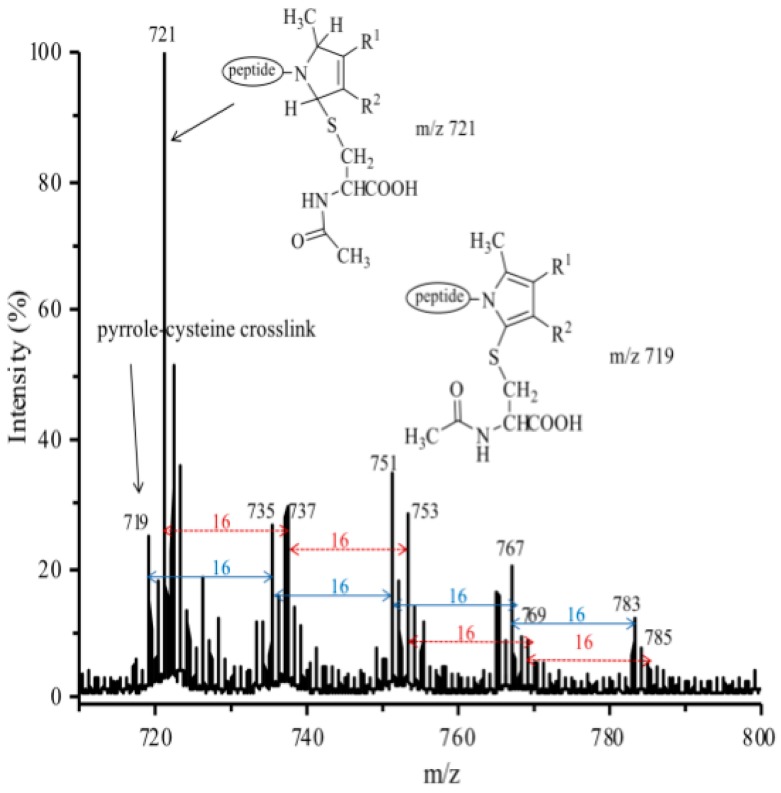
MALDI-TOF MS of pure iso[4]LGE_2_-pyrrole reaction with N-acetylcysteine under air at 25 °C for 8 days. A series of peaks that differ in m/z by 16 show the addition of oxygen atoms.

To test the possibility that nucleophilic thiols can participate in isoLG-derived pyrrole-mediated protein crosslinking, HPLC-purified iso[4]LGE_2_-pyrrole (**3**) was incubated in the presence of ten equivalents of N-acetylcysteine (**4**) under air at 25 °C for several days (Scheme 4). Representative MALDI-TOF spectra of the reaction mixture are shown in Figure 3. In the presence of N-acetylcysteine, incubation of iso[4]LGE_2_-pyrrole at a neutral pH under air showed no pyrrole–pyrrole crosslinking. Thus, the thiol-containing compound inhibited the crosslinking of iso[4]LGE_2_-pyrrole (Figure 4). Instead a series of new ions was produced. They corresponded to pyrrole-cysteine adducts and a series of derivatives that were produced from them by the addition of one or more atoms of oxygen (Figure 5). The amount of these more oxygenated products increased with longer incubation. A thiol-pyrrole conjugate was detected by MALDI-TOF MS that presumably incorporates a bond between the pyrrole ring at C-2 to the sulfur atom of the N-acetylcysteine (see **5** in Scheme 3) [10,11]. Thus, thiols inhibit the pyrrole dimerization presumably by intercepting pyrrole cation radicals generated by electron transfer from the pyrrole to oxygen.

### 3.4. Although Initially Slow, the Oxidation of Pure Iso[4]LGE_2_-Pyrrole with Air Accelerates. 

**Figure 6:**
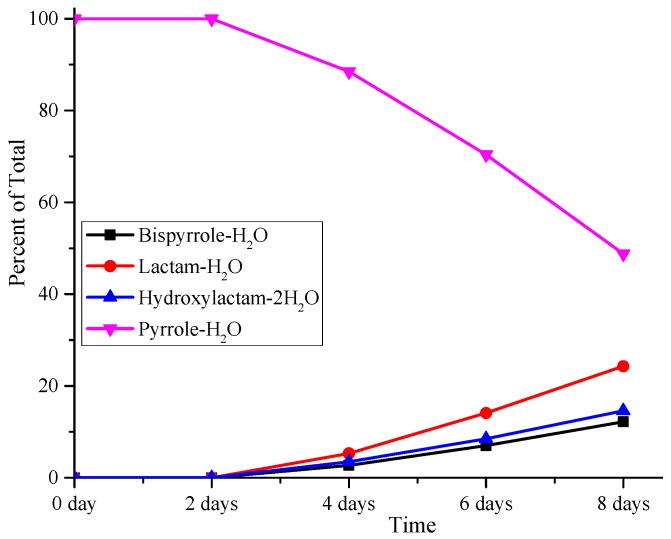
Time course of the oxidative reactions of pure iso[4]LGE_2_-pyrrole under air at 25 °C for 8 days. As shown in Supplementary Information Figure S3, the observed peak-intensity for pyrrole-H_2_O is 100 (for each time point). Peak intensity of bispyrrole-H_2_O, lactam-H_2_O, and hydroxylactam-2H_2_O was zero for the first two days, and then gradually increased. The total intensity is the sum of pyrrole-H_2_O, bispyrrole-H_2_O, lactam-H_2_O, and hydroxylactam-2H_2_O. The peak intensity of each product is divided by the total intensity to provide the percent of the dtotal shown in the figure.

**Figure 7:**
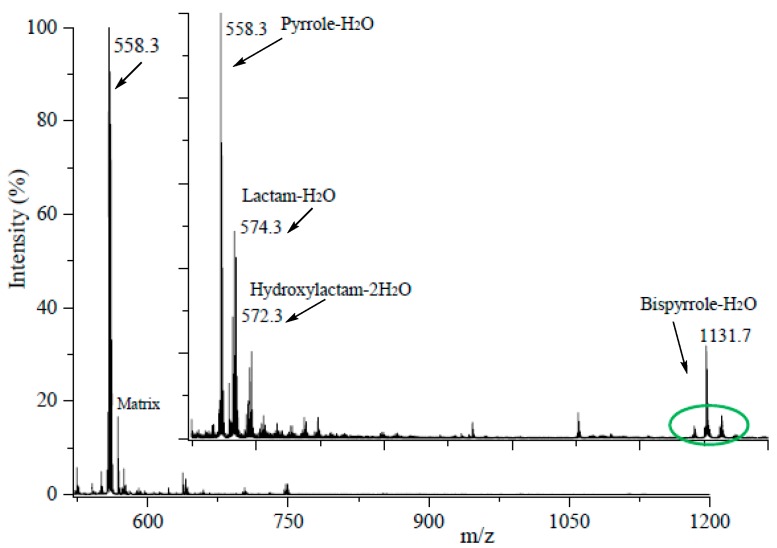
MALDI-TOF MS of pure iso[4]LGE_2_-pyrrole (**3**) and its oxidation products: lower trace = pure iso[4]LGE_2_ -pyrrole (**3**), upper trace = after incubation under air at 25 °C for 8 days.

The reaction time course (Figure 6) for incubation of pure iso[4]LGE_2_-pyrrole under air at 25 °C in aqueous acetonitrile solution (10% acetonitrile/H_2_O) was monitored for eight days (see Supplementary Information Figure S3). HPLC-purified pyrrole initially exhibited little proclivity toward oxidation or dimerization when exposed to air. However, eventually the generation of these products accelerated (Figure 6). MALDI-TOF mass spectra of pure iso[4]LGE_2_-pyrrole and the reaction product mixture after exposure to air for eight days are shown in Figure 7. During the first two days, almost no oxidized product was formed. At longer reaction times, lactam, hydroxylactam, and bispyrrole all became prominent. 

**Scheme 5:**
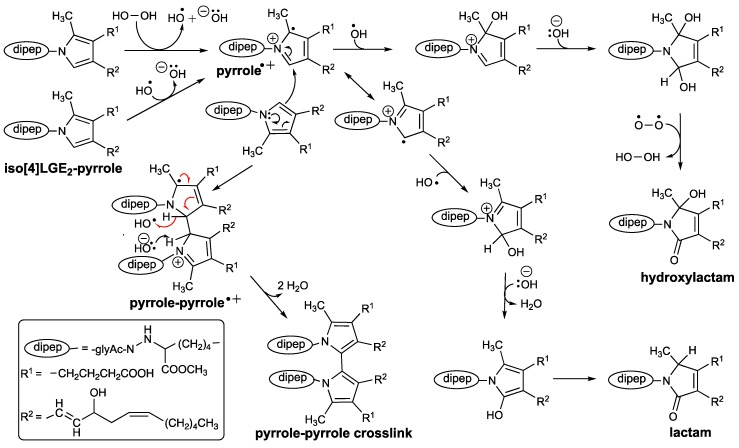
A mechanism involving single electron transfer from iso[4]LGE_2_-pyrrole to hydrogen peroxide for pyrrole–pyrrole oxidative coupling and oxidation to lactam and hydroxylactam derivatives. Single electron movement is indicated with red half-headed arrows.

We postulated that the eventual acceleration of the oxidative consumption of iso[4]LGE_2_-pyrrole (**3**) is explicable in terms of a change in mechanism. Initially, oxygen serves as the acceptor of an electron from the pyrrole (Scheme 3). Hydrogen peroxide is a byproduct of the oxidation. Consequently, the pyrrole can also transfer an electron to hydrogen peroxide, producing a hydroxide anion and a hydroxyl radical. The change of mechanism to a single electron transfer from pyrrole to hydrogen peroxide instead of to oxygen is expected to accelerate the oxidative transformations of the pyrrole because the electron transfer to hydrogen peroxide is faster than to oxygen [12]. Hydroxyl radical-pyrrole cation radical combination can produce iminium intermediates that add hydroxide to produce a diol intermediate, or lose a proton leading to lactam. Additional mechanistic complexity might involve electron transfer from a second molecule of pyrrole to the hydroxyl radical to produce an additional pyrrole cation radical (Scheme 5). 

### 3.5. Autoxidation of Iso[4]Lge_2_-Pyrrole is Promoted by Single Electron Transfer Catalysts. 

**Figure 8:**
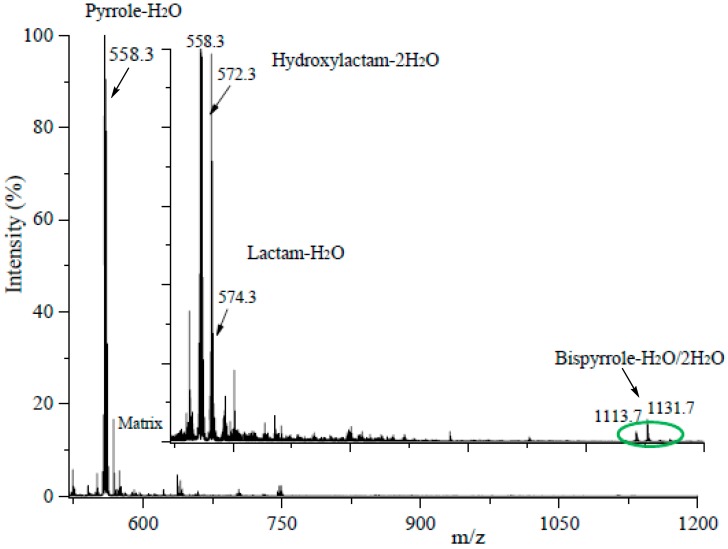
MALDI-TOF MS of pure iso[4]LGE_2_-pyrrole and its oxidation products: lower trace = pure iso[4]LGE_2_-pyrrole, upper trace = after exposure to 2 mol% APS and 1 mol% TEMED at 37 °C for 2 h.

Contaminants in the reaction mixture of iso[4]LGE_2_ with N-acetyl-gly-lys promoted rapid oxidative transformations of iso[4]LGE_2_-pyrrole. Therefore, we tested various potential catalysts of pyrrole oxidation for their ability to promote pyrrole–pyrrole oxidative coupling and oxidation of iso[4]LGE_2_-pyrrole to lactams and hydroxylactams by air. Persulfate ion promotes pyrrole polymerization by a single electron transfer mechanism involving the rate-determining generation of a pyrrole radical cation intermediate [13]. Incubation of the isoLG-pyrrole **3** with 1–2 mol% ammonium persulfate (APS, K_2_S_2_O_8_) and 0.5–1 mol% tetramethylethylenediamine (TEMED) for 2 h generated a product mixture containing lactam and hydroxylactam, but only small amounts of pyrrole–pyrrole dimer (Figure 8). 

**Figure 9:**
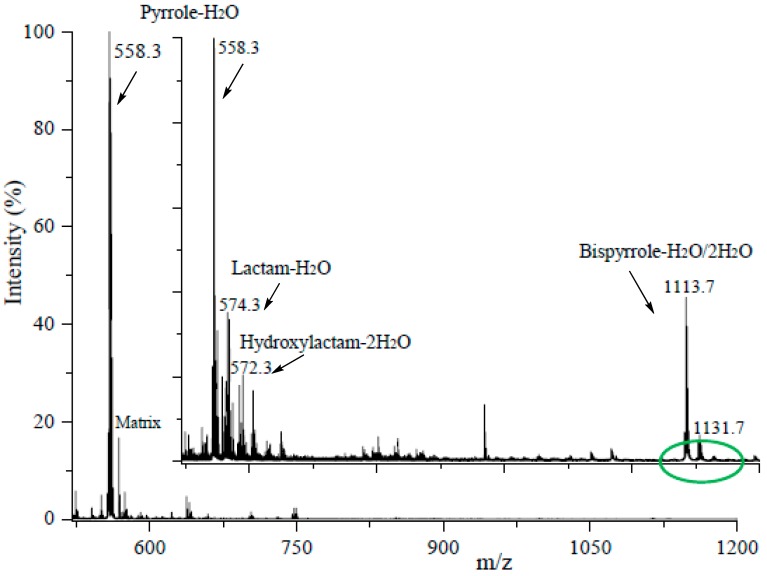
MALDI-TOF MS of pure iso[4]LGE_2_-pyrrole and its oxidation products: lower trace = pure iso[4]LGE_2_-pyrrole, upper trace = after exposure to 10 mol% TEMPO at 37 °C for 3 h.

Nitroxide radicals, especially (2,2,6,6-tetramethylpiperidin-1-yl)oxyl [14,15,16], are stable, persistent free radicals [17]. Tetramethylpyrrolidine-N-oxide (TEMPO) can function as a single electron transfer oxidant [18,19]. We tested its ability to promote isoLG-pyrrole dimerization. Treatment of the isoLG-pyrrole **3** with 10 mol% of TEMPO at 37 °C for 3 h cleanly generated a product mixture containing similar amounts of lactam and pyrrole–pyrrole dimer, and the products were not degraded by longer incubation or the use of a larger mol% of TEMPO. Thus, pyrrole–pyrrole crosslinking of isoLG-pyrrole is facilitated by TEMPO (Figure 9).

Although not a stable free radical, trimethylamine-N-oxide (TMAO) proved similarly effective to TEMPO in promoting oxidation of the isoLG-pyrrole derivative **3** of N-acetyl-gly-lys methyl ester (to lactam and hydroxylactam) and oxidative pyrrole–pyrrole coupling. Treatment of HPLC-purified pyrrole with 10 mol% of TMAO at 37 °C for 3 h cleanly generated a product mixture containing similar amounts of lactam and pyrrole–pyrrole dimer (see Supplemental Information Figure S4), and the products were not degraded by longer incubation or in the presence of a larger mol% of TMAO. 

## 4. Discussion 

Pure Isolg Pyrroles are Unreactive toward Oxygen

Previously, the reaction of phosphatidyl­ethanolamine (PE) with an isoLG was reported to produce a stable pyrrole derivative that resists further oxidation to lactams [20]. This stability was ascribed to lower concentrations of O_2_ in organic solvents, such as CHCl_3_ (in which the reaction of PE with isoLGs was conducted), than in H_2_O (the solvent used in the current study of the reaction of proteins and the N-acetyl-lys-gly methyl ester dipeptide with isoLGs). However, the opposite was found to be the case, the concentration of oxygen in CHCl_3_ was higher than in air-saturated H_2_O. The concentration O_2_ in air-equilibrated organic solvents such as CHCl_3_ (2.05 mM), acetone (2.4 mM), acetonitrile (2.42 mM), or ethanol (1.94 mM) is about seven-fold higher than in air-equilibrated H_2_O (0.28 mM) [21,22,23,24,25,26,27]. Given the susceptibility of the autoxidation of the N-acetyl-lys-gly methyl ester dipeptide-derived isoLG pyrrole to catalysis, the stability of PE-isoLG pyrrole may be a consequence of the absence in CHCl_3_ of catalytic impurities that promote its autoxidation, such as traces of redox active metal ions that are soluble in water but not chloroform. Redox active metal ions can be reduced by single electron transfer and then reoxidized by oxygen. This chemistry may contribute to isoLG-pyrrole oxidation and oxidative coupling in vivo, especially under conditions where elevated levels of redox active metal ions, e.g., Cu^+n^ or Fe^+n^, accumulate.

Single Electron Transfer Initiates Oxidation and Oxidative Coupling of IsoLG-pyrroles

The present study showed that the reaction of pure isoLG-pyrroles with oxygen is initially slow, but after incubation under air for two days, a faster reaction with t_1/2_ = 6 days eventually occurs. The initial generation of a pyrrole cation radical intermediate and superoxide by electron transfer to oxygen (Scheme 3) is supported by the formation of N-acetylcysteine adducts and the suppression of dimer production when the isoLG-pyrrole is exposed to oxygen in the presence of N-acetylcysteine. Thus, incubation of iso[4]LGE_2_-pyrrole with N-acetylcysteine at a neutral pH under air showed no pyrrole-to-pyrrole crosslinking. Instead, a series of new ions was produced corresponding to pyrrole N-acetylcysteine adducts and a series of derivatives were produced from them by the addition of one or more atoms of oxygen. Presumably, the thiol nucleophile inhibits pyrrole dimerization by intercepting the electrophilic pyrrole cation radical. Furthermore, the oxidative transformations of isoLG-pyrrole are greatly accelerated by the single electron transfer catalyst TEMPO (Figure 9). Hydrogen peroxide is a likely byproduct of oxidative pyrrole–pyrrole coupling. However, hydrogen peroxide is not expected to accumulate because hydrogen peroxide is a superior electron acceptor compared to molecular oxygen. A change in mechanism, involving single electron transfer to hydrogen peroxide (Scheme 5) for the uncatalyzed reaction with air, may account for the eventual acceleration. 

TMAO Promotes Oxidation and Oxidative Coupling of IsoLG-Pyrrole

It seems likely that the oxidative transformations of isoLG-pyrroles in vivo will involve catalysis. The accumulation of trimethylamine-N-oxide (TMAO) through gut microbial metabolism of phosphatidylcholines [28,29] and L-carnitine [30] is strongly positively correlated with risk of cardiovascular disease [6]. TMAO can act as a prooxidant in the autooxidation of methyl linoleate [31]. Because understanding the molecular mechanisms of TMAO-induced oxidative transformations of biomolecules is likely to provide important insights into its pathological involvements, we tested its effect on the oxidative transformations of isoLG-pyrrole. It proved similarly effective to TEMPO in promoting oxidation and oxidative coupling of isoLG-pyrrole. It is conceivable that this chemistry contributes to the pathological consequences of TMAO. Further mechanistic investigation of TMAO-induced isoLG pyrrole oxidation reactions may provide insights into the chemistry involved in pathologies associated with elevated levels of TMAO.

## 5. Conclusions

In summary, the present study established that, (1) pure isoLG pyrroles are inherently unreactive toward oxidation by air, but are susceptible to an autocatalytic acceleration of the reaction, (2) an electrophilic cation radical produced by electron transfer from an electron-rich isoLG-pyrrole to oxygen can be intercepted by the nucleophilic thiol of N-acetylcysteine, and (3) oxidative transformations of isoLG pyrroles are promoted by single electron transfer catalysts. IsoLGs generated through the cyclooxygenase pathway accelerate the formation of the type of oligomers of amyloid beta that have been associated with neurotoxicity [5]. If that oligomerization involves oxidative coupling of isoLG pyrroles, it is now expected to be prevented under the anoxic in vitro conditions established in the present study. Mechanistic understanding of isoLG-induced protein crosslinking is needed as a basis for the development of therapeutic counter measures. Rapid adduction of isoLGs with nucleophiles has precluded their isolation from the biological environment of their generation. IsoLG-derived pyrrole derivatives of primary amino groups of biomolecules, e.g., proteins or ethanolamine phospholipids, are susceptible to oxidation and oxidative coupling. Stable end products, e.g., lactams and hydroxylactams, have been characterized. Although their exceptional ability to promote protein-protein crosslinking has been recognized for decades, the present study provides the first molecular level insights into the process and uncovered the first example of the oxidative coupling of an isoLG-derived pyrrole intermediate with N-acetylcysteine. Similar adduction of glutathione (GSH) might serve to block isoLG-induced protein-protein crosslinking in vivo.

Further studies are required to provide mechanistic understanding of the observations that (1) the reaction of oxygen with isoLG pyrroles gradually accelerates, (2) further oxygen atoms are added to the initial products, and (3) TMAO catalyzes the reactions of isoLG pyrroles with oxygen. Because electron transfer to hydrogen peroxide is faster than to oxygen [12], one possible explanation for acceleration of the reaction of isoLG pyrroles with air is that an alternative pathway involving electron transfer to hydrogen peroxide, produced by the reduction of oxygen, becomes rate-determining. Future studies should also explore the possibility that other catalysts, likely to be encountered in vivo, might include redox active metal ions, while antioxidant enzymes may be effective in preventing crosslinking by intercepting reactive intermediates, e.g., superoxide by superoxide dismutase, or hydrogen peroxide by catalase.

## Supplementary Materials

The following are available online at https://www.mdpi.com/2571-5135/8/2/12/s1, Table S1. Optimized parameters for triple quadrupole mass spectrometer. Table S2. Optimized parameters for MALDI-TOF mass spectrometer. Figure S1. MALDI-TOF spectra of iso[4]LGE_2_-pyrrole. Figure S2. MALDI-TOF spectra of iso[4]LGE_2_ and acetyl-Gly-Lys-O-methyl ester exhibiting new peaks not present in the matrix. Figure S3. MALDI-TOF spectra of reaction mixtures produced upon incubation of the HPLC-purified iso[4]LGE_2_ pyrrole derivative of acetyl-Gly-Lys-O-methyl ester showing the evolution of peaks not present in the matrix. Figure S4. MALDI-TOF spectrum of purified 1 mM iso[4]LGE_2_-pyrrole autoxidation reaction product mixture generated after 3 h incubation at 37 ℃ in the presence of 1 mM TMAO.
