# Supplementary material for: The Adductomics of Isolevuglandins: Oxidation of IsoLG Pyrrole Intermediates Generates Pyrrole–Pyrrole Crosslinks and Lactams
Source: High Throughput. 2019 May 10;8(2):12. doi: 10.3390/ht8020012 (PMC6630840; doi:10.3390/ht8020012)
Supplement: Supplementary file 1 [file high-throughput-08-00012-s001.pdf]

Supplementary Information for: The Adductomics of Isolevuglandins: Oxidation of Pyrrole Intermediates Generates Pyrrole-Pyrrole Crosslinks and Lactams

Wenxhao Bi, Geeng-Fu Jang, Lei Zhang, John W. Crabb, James Laird, Mikhail Linetsky and Robert G. Salomon

Item	Pages
**Table S1.** Optimized parameters for triple quadrupole mass spectrometer.	1
**Table S2.** Optimized parameters for MALDI-TOF mass spectrometer.	1
**Figure S1.** MALDI-TOF spectra that do not exhibit peaks that correspond to iso[4]LGE$_2$ and acetyl-gly-lys-o-methyl ester. The positions where the missing peaks would appear are indicated with red arrows in the left panel.	2
**Figure S2.** MALDI-TOF spectra of iso[4]LGE$_2$ and acetyl-gly-lys-o-methyl ester exhibiting new peaks not present in the matrix.	2
**Figure S3.** MALDI-TOF spectra of hplc purified iso[4]LGE$_2$ pyrrole derivative of acetyl-gly-lys-o-methyl ester and of reaction mixtures produced upon incubation under air for 2, 6, and 8 days showing the evolution of peaks corresponding to oxidized pyrrole, i.e., lactam and hydroxylactam, and bispyrrole.	3
**Figure S4.** MALDI-TOF spectrum of purified 1 mm iso[4]LGE$_2$-pyrrole autoxidation reaction product mixture generated after 3 h incubation at 37 °C in the presence of 1 mm tmao showing an abundance of bispyrrole in contrast to its absence after 2 days incubation in the absence of TMAO shown in Figure S3.	4

Table 1. Optimized parameters for triple quadrupole mass spectrometer.

Parameters	Data
Declustering Potential (DP)	30
Focus Potential (FP)	250
Entrance Potential (EP)	10
Nebulizer Gas (NEB)	10
Curtain Gas (CUR)	8
Ion Spray Voltage (IS)	4000
Temperature (TEM)	200

Table 2. Optimized parameters for MALDI-TOF mass spectrometer.

Acquisition Method	Parameters	Data	Parameters	Data
	Shots/sub-spectrums	50	Bin size (ns)	0.5
	Total shots/spectrum	1000	Input bandwidth (MHZ)	500
	Laser intensity	3000	Detector voltage multiplier	0.92
	Vertical scale (v)	0.5	Final detector voltage (KV)	2.015
Processing Method	Calibration Parameters	Data	Peak Detection Parameters	Data
	Min S/N	20	Min S/N	10
	Mass tolerance (m/z)	2	Local noise window width (m/z)	250
	Min peaks to match	4	Min peak width (bins)	2.9
	Max error (ppm)	100	Mass resolution	22000

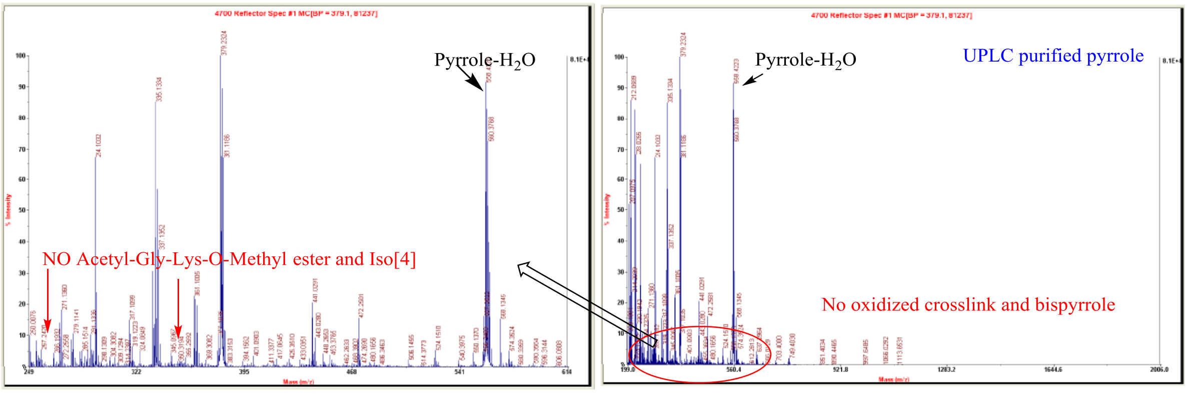

Figure S1. MALDI-TOF spectra that do not exhibit peaks that correspond to iso[4]LGE$_2$ and acetyl-gly-lys-o-methyl ester. The positions where the missing peaks would appear are indicated with red arrows in the left panel.

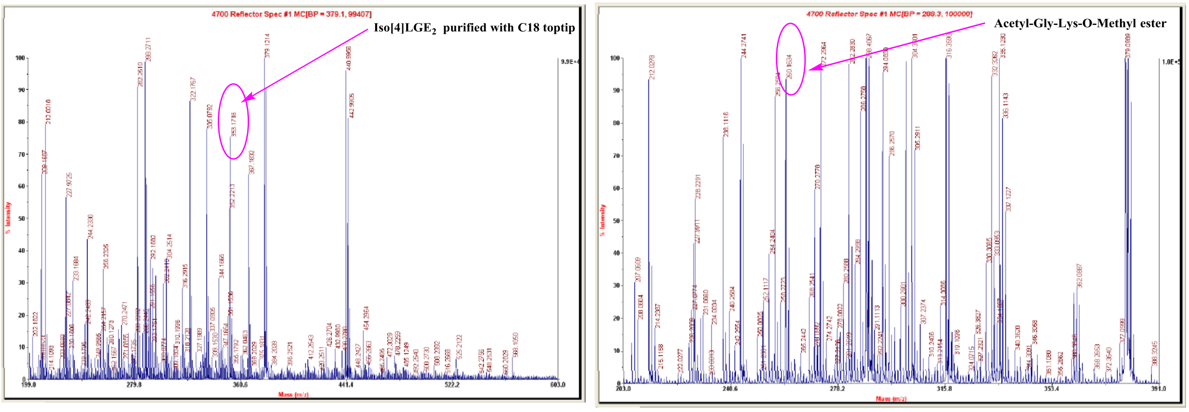

Figure S2. MALDI-TOF spectra exhibiting peaks (indicated with arrows) not present in the matrix that correspond to iso[4]LGE$_2$ and acetyl-gly-lys-o-methyl ester. These peaks are absent in Figure S1.

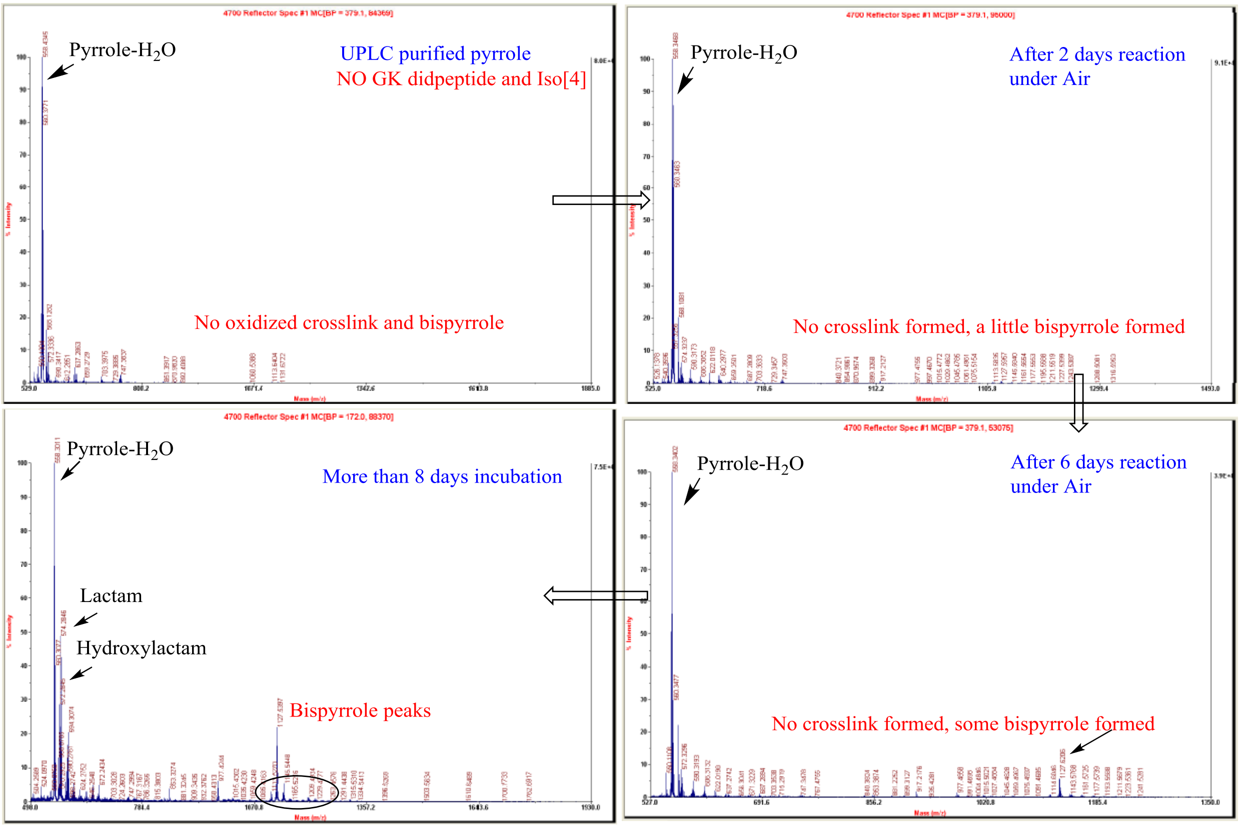

Figure 3. MALDI-TOF spectra of HPLC purified iso[4]LGE2 pyrrole derivative of acetyl-Gly-Lys-O-methyl ester and of reaction mixtures produced upon incubation under air for 2, 6, and 8 days showing the evolution of peaks corresponding to oxidized pyrrole, i.e., lactam and hydroxylactam, and bispyrrole.

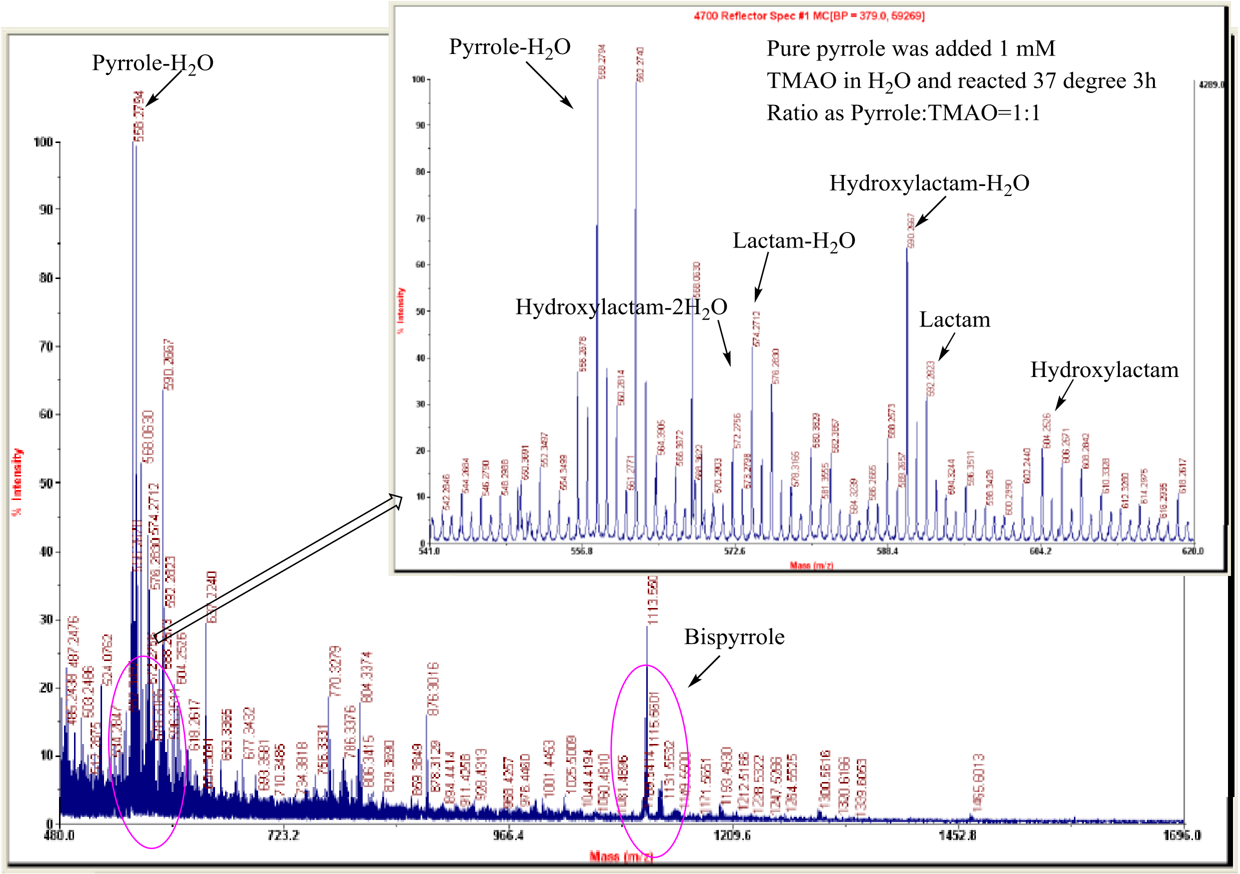

Figure S4. MALDI-TOF spectrum of purified 1 mM iso[4]LGE$_2$-pyrrole autoxidation reaction product mixture generated after 3 h incubation at 37 ℃ in the presence of 1 mM TMAO showing an abundance of bispyrrole in contrast to its absence after 2 days incubation in the absence of TMAO shown in Figure S3.
